# Free Thyroxine Concentrations Moderate the Response to a Cognitive Remediation Therapy in People With Early Psychosis: A Pilot Randomized Clinical Trial

**DOI:** 10.3389/fpsyt.2020.00636

**Published:** 2020-07-07

**Authors:** Francesc Estrada, Josep Maria Crosas, Maribel Ahuir, Sara Pérez-Muñoz, Wanda Zabala, Raquel Aguayo, Juan David Barbero, Itziar Montalvo, Meritxell Tost, Laura Llauradó, Armand Guardia, Diego Palao, José Antonio Monreal, Javier Labad

**Affiliations:** ^1^ Department of Mental Health, Institut d’Investigació i Innovació Parc Taulí (I3PT), Parc Taulí Hospital Universitari, Sabadell, Spain; ^2^ Department of Psychiatry and Legal Medicine, Universitat Autònoma de Barcelona, Barcelona, Spain; ^3^ Centro de Investigación Biomédica en Red de Salud Mental (CIBERSAM), Carlos III Health Institute, Madrid, Spain

**Keywords:** cognitive rehabilitation, memory, psychosis, thyroid, cognition

## Abstract

**Introduction:**

Cognitive deficits are a cause of functional disability in psychotic disorders. Cognitive remediation therapy (CRT) might be applied to improve these deficits. We conducted a pilot study to explore whether thyroid hormones might predict the response to CRT in patients with recent-onset psychosis (ROP).

**Methods:**

Twenty-eight stable ROP outpatients (9 women) were randomized to receive computerized CRT (N=14) or treatment as usual (TAU) (N=14), over three months. Both cognitive and thyroid functions were assessed at the baseline and after those three months to all patients. A full cognitive battery (CANTAB) was administered before and after the treatment. Serum levels of thyroid-stimulating hormone (TSH) and free thyroxine (FT4) were measured. FT4 concentrations were recoded into a dichotomic variable (FT4 group) based on the median of the sample (1.2 ng/dL). Data were analyzed on an intention-to-treat basis with linear mixed models. Afterwards, we offered CRT to all participants from the TAU group and seven enrolled CRT, reassessing them when finished. Secondary analyses were repeated in a sample of 14 participants who completed the CRT (either from the beginning or after the TAU period) and attended at least one third of the sessions.

**Results:**

The linear mixed models showed a significant time x CRT x FT4 group effect in two cognitive tasks dealing with executive functions and sustained attention (participants with higher FT4 concentrations worsened executive functions but improved sustained attention after CRT). In the secondary analysis including all patients assigned to CRT, higher FT4 concentrations were associated with a poorer response in verbal memory but a better response in spatial working memory.

**Conclusions:**

Free thyroxine concentrations moderate the response to a CRT in patients with early psychosis.

## Introduction

Cognitive dysfunction is a core feature of schizophrenia experienced by approximately 75% of the patients, and it may occur prior to the onset of psychosis, as it is usually unaltered despite positive symptom remission (1). Actually, cognitive function at psychosis onset is what most strongly predicts worse long-term overall functioning as well as worse functionality in social and occupational areas (2, 3).

Without any specific medication approved to treat cognitive impairment (4), there has been an increased interest in the efficacy of cognitive remediation therapy (CRT) in the last decade. CRT is an intervention targeting cognitive deficit using scientific principles of learning with the ultimate goal of improving functional outcomes (5). It has been shown to have a significant impact on cognition with a medium effect size: 0.41 in the meta-analysis of McGurk et al., and 0.45 in the one from Wykes (5, 6). More specifically, some studies have shown improvements in early psychosis patients reporting moderate to large effects on cognitive outcomes, such as verbal and working memory, visuospatial ability, psychosocial functioning, problem solving, or executive functioning (7, 8). Moreover, in those patients, a significant functional benefit such as in employment and in social functioning has been found (9).

However, the impact on symptoms is almost null, as it is more significant in less symptomatic patients (5). In fact, cognitive deficits appear to reduce the potential benefit of rehabilitation programmes, contributing to higher rates of institutionalization (10). Nonetheless, Cella et al. showed a significant effect on negative symptoms (0.36) in established schizophrenia patients (11). More recently, Ventura et al. found an improvement on negative symptoms at 12 months in the first episode of schizophrenia patients (FEP), comparing CRT to healthy behaviour training (12).

It is important to remark that the effect of CRT appears to be homogeneous regardless of the method used and the programme duration (5, 13); however, a study has highlighted the importance of adjusting the level of computerized exercises to the patients’ cognitive performances (14). Nevertheless, it seems that even with limited duration of the intervention (5–15 h), mild improvement can be observed (15).

Although CRT is an effective approach, there are data showing that 25–44% of the participants receiving this intervention will not improve (5, 16, 17). To date, only a limited number of studies have considered how individual characteristics may affect treatment response. Some of them have suggested that being younger (18, 19), reporting fewer symptoms (5) and having more severe cognitive difficulties (7, 20) can contribute to benefiting more from CRT. However, it seems to be limited converging evidence, and the data are sometimes contradictory. A recent review (21) has explored 18 potential moderators to have an effect on CRT; nonetheless, they found no high-quality replicated evidence for any of them.

To design a tailored treatment, searching for biomarkers as moderators or mediators of cognitive improvement with CRT in schizophrenia is an emerging field of research. First, some studies have studied several candidate genes and proteins that could predict cognitive improvement after CRT such as catechol-o-methyltransferase (COMT) (22–24), brain-derived neurotrophic factor (BDNF) (25, 26), or excitatory amino acid transporter 2 (EAAT2) (27). Neuroimaging studies suggest that cortical reserve, defined as grey matter volume (28), cortical thickness (29), and integrity of the right fronto-occipital fasciculus, right corticospinal tract and bilateral medial lemnisci (30) can moderate the response to CRT in cognitive domains such as social cognition, verbal and non-verbal memory, attention/vigilance, and executive function. However, two recent reviews have concluded that specific biological pathways underlying cognitive remediation are still unclear, and the authors could not provide any guidance on clinical decision-making in this field (21, 31).

Another potential biomarker moderating the response to CRT could be thyroid hormones, as they play an important role in cognitive performance in psychiatric and non-psychiatric populations (32). Thyroid hormones participate in brain development with effects on actin polymerization, microfilament organization, and neuronal migration (33). Hence, individuals with overt hypothyroidism have mild to moderate impairment in cognition, especially in memory; those with overt thyrotoxicosis may have reduced attention/concentration and executive function (34), but these symptoms are usually reversible with levo-thyroxine (L-T4) treatment?. Furthermore, in chronic patients with schizophrenia, several thyroid test abnormalities have been described: decreased activity of the hypothalamic–pituitary–thyroid axis, euthyroidism with a low triiodothyroxine (T3) levels, and increased thyroid antibodies (35), where the free T3 (FT3) levels are associated with better cognitive function as assessed by the Mini-Mental State Examination (MMSE) (36). More specifically, our group has studied the cognitive function in early psychotic patients and the potential contribution of thyroid hormones. Barbero et al. found that better cognitive performance in the attention/vigilance domain of the MATRICS Consensus Cognitive Battery (MCCB) was associated with subtly increased free thyroxine (FT4) levels (37). In addition, the same group performed a 1-year follow-up study that reported a U-shaped relationship between FT4 levels and longitudinal cognitive changes. In other words, the highest and lowest FT4 values were associated with worsened attention in the follow-up assessment, suggesting that subtle differences in normal FT4 levels may play a role in the attention domain in early psychotic patients (38). However, other studies had reported previously that high levels of FT3 were associated with poorer performance on executive functioning and processing speed with no association of FT4 with cognitive domains (39).

To our knowledge, there are no studies exploring the role of thyroid function as a predictor of cognitive improvement after CRT. Thus, we aimed to conduct a pilot study to explore whether thyroid hormone levels, particularly FT4, might moderate treatment response to a CRT when compared to treatment as usual (TAU). We also aimed to study in what degree FT4 levels predict longitudinal cognitive changes in the subsample of patients completing at least one third of CRT sessions (excluding those who did not engage in the therapy).

## Materials and Methods

### Participants

We studied a population of 28 patients (9 women) with a recent-onset psychosis (ROP) aged between 18 and 40 years old and with less than 3 years of duration of the illness. Our sample is a convenience sample of patients who were consecutively recruited from the Early Intervention Service from Hospital Universitari Parc Taulí Sabadell, (Spain). All participants were outpatients with stable illness [< 4 points in each positive item of the Positive and Negative Syndrome Scale (PANSS) and being on community treatment for at least 4 weeks]. The included patients with early psychosis fulfilled the DSM-IV-TR criteria for schizophrenia (n=17), schizoaffective disorder (n=8), psychotic disorder not otherwise specified (NOS) (n=2) or bipolar disorder (n=1). The exclusion criteria were refusal to participate, severe neurological disease or intellectual disability, pregnancy, substance-induced psychosis, history of severe traumatic brain injury, and visual deficits or language difficulties that could influence cognitive assessment and intervention.

Ethical approval was granted by the Drug Research Ethics Committee (CEIm) from Parc Taulí Hospital Universitari. All participants received extended information about the study, and written informed consent was obtained. The biological samples and the clinical data were collected following the Spanish legislation of data protection to ensure the confidentiality of the patients.

### Design

A randomized crossover clinical trial was performed as a pilot study (NCT04418570). In a first step, participants were randomized into two groups: CRT (n=14) or TAU (n=14) in the Early Intervention Service. Participants included in the CRT arm received the same health care in the Early Intervention Service as those participants included in the TAU group. All patients were assessed (clinical and cognitive assessment) and a blood sample was obtained at baseline and after the CRT/TAU period, 3 months later. We conducted an intention-to-treat analysis for cognitive changes over the first three months (1^st^ wave).

Afterwards, patients were offered to switch arms to give access to the intervention to the TAU group. At that point, only 7 from the 14 patients who were in TAU finally enrolled the CRT (2^nd^wave). Participants were reassessed with the same cognitive battery three months later (after the CRT). We conducted per-protocol analyses by excluding patients who did not complete at least one third of the CRT sessions. Therefore, we finally had a sample of 14 patients: 7 patients from the initial CRT group (1^st^ wave) and 7 patients added in the 2^nd^ wave.

The flow chart of the participants of the clinical trial is depicted in **Figure S1** from the **Supplementary Material**.

### Intervention

The intervention consisted of a computerized CRT through Neuropersonal Trainer (NPT) software (40). This CRT includes two rehabilitation modules: 1) the cognition module, that addresses sustained, selective, and divided attention; verbal, spatial, visual and working memory; and executive functions including inhibition, sequence, and planning; 2) the social cognition module (41), which allows working different aspects of emotional processing, the theory of mind and cognitive biases through 43 multimedia-based tasks. Both modules have different levels of complexity.

All exercises offered immediate success/failure feedback and a final summary of the results performance. Each rehabilitation session with NPT was individualized to the cognitive needs of each patient by an expert neuropsychologist, who adjusted the difficulty level in each treatment session based on the participant baseline cognitive profile and his/her task performance in previous treatment sessions. The neuropsychologist was always guiding the sessions, assuring the understanding of the different tasks, and individually coaching every patient to achieve the correct performance.

The training was administered in 1.5 h sessions, twice a week, during 12 weeks (36 h of total duration). To assure a therapeutic effect, a minimum of 12 h of training for each participant was established by clinical consensus.

### Screening Visit

All patients were interviewed by a psychiatrist, and the OPCRIT checklist version 4.0 (available at http://sgdp.iop.kcl.ac.uk/opcrit) was used to obtain DSM-IV diagnoses. PANSS was used to assess the severity of psychotic symptoms (42). The Calgary Depression Scale (CDS) was administered to assess depressive symptoms (43). The Personal and Social Performance scale (PSP) was used to measure functionality (44). These scales were administered by a psychiatrist or clinical psychologist with experience in the treatment of patients with psychotic disorders.

Socio-demographic data, clinical variables, and substance use in the last month were obtained by a semi-structured interview, to assure that the two randomized groups were comparable at baseline assessment. Alcohol use was measured by standard Spanish units/day, tobacco use was measured by cigarettes/day, and cannabis use was measured by qualitative description (daily or sporadic).

All patients received antipsychotic treatment. Overall, 13 were on LAI treatment (11 with paliperidone and 2 with aripiprazole). The rest were treated with oral antipsychotics: aripiprazole (n=8), clozapine (n=3), paliperidone (n=2), risperidone (n=1), and olanzapine (n=1). Each antipsychotic dose was converted to chlorpromazine equivalents in mg/day (45). We considered long-acting aripiprazole 400 mg as 267 mg/day of chlorpromazine.

### Cognitive Assessment

To assess the cognitive function, we utilized the Cambridge Neuropsychological Test Automated Battery (CANTAB) for schizophrenia (46). This battery has 8 specific tests to assess cognition in patients with psychosis. Those are: Attention Switching Task (AST; assessing attention and response latencies); One Touch Stockings of Cambridge (OTS; assessing executive functioning: spatial planning and working memory); Paired Associates Learning (PAL; assessing visual memory and new learning); Reaction Time (RTI; assessing reaction time: motor and mental response speeds, as well as measures of movement time, reaction time, response accuracy and impulsivity); Rapid Visual Information Processing (RVP; assessing sustained attention); Emotion Recognition Task (ERT; assessing theory of mind processes); Spatial Working Memory (SWM; assessing spatial working memory: retention and manipulation of visuospatial information, strategy and working memory errors); Verbal Recognition Memory (VRM; assessing verbal memory and new learning). The description of all different cognitive tasks is included in List S1 from the **Supplementary Material**.

### Hormonal Assessment

A fasting blood sample was obtained in the morning between 8 and 10 a.m.to determine thyroid hormones. Serum levels of thyroid-stimulating hormone (TSH) and FT4 were measured using an electrochemiluminescence immunoassay on an automated analyser (Cobas 8000 e801, Roche Diagnostics, Mannheim, Germany).

### Statistical Analysis

The statistical analysis was conducted with SPSS version 22.0 (IBM, USA). To assess the differences on baseline variables between two groups (TAU and CRT), we used Student’s t-test for continuous variables, the Mann-Whitney U test for ordinal variables, and a chi-squared test for categorical variables. We considered significance to be a p-value <0.05 (two-sided).

Longitudinal changes in cognitive variables were analyzed with linear mixed models. We previously transformed cognitive scores into z-scores. Z-scores are linearly transformed data values having a mean of zero and a standard deviation of 1. We also divided all participants into two groups for FT4 concentrations taking into account the median of the baseline measures (1.2 ng/dL): 1) high FT4 (≥ 1.2 ng/dL) and 2) low FT4 (< 1.2 ng/dL).

In the first analyses (intention-to-treat basis, 1^st^ wave), intervention (CRT vs TAU) was defined as a factor, being the TAU arm as the reference category. Time (visit) and FT4 group were also considered factors (baseline visit and low FT4 were set as the reference categories). TSH concentrations (z-scores) were used as a continuous covariate for adjustment. The following fixed effects were tested: time (baseline vs 3-months visit), intervention (CRT vs TAU), FT4 (low vs high) and TSH. The following interactions were also tested: time x intervention, time x FT4, intervention x FT4, time x intervention x FT4. Participants were considered random effects in the model. Restricted Maximum Likelihood (REML) was used for fitting the model and estimating the effects of the variables included in the model. First-order autoregressive was used as the structure for the within-subject covariance matrix.

In the second analyses (per-protocol, including all participants completing at least one third of the CRT sessions during the 1^st^ wave or the 2^nd^ wave), the following fixed effects were considered: time, FT4 group, TSH, time x FT4 group. Participants were also considered random effects in the model.

Our clinical trial was considered a pilot study. Some authors have suggested the role of thumb of 12 per group for a pilot study when there is no prior information (47). As we had no previous data on the moderation of FT4 concentrations on cognitive changes, we decided to include a sample size of 28. This sample size would be sufficient to detect moderate to large effects with a statistical power of 90% and two-sided 5% significance (48). However, it is important to underscore that our study is underpowered for detecting small effects.

Although we conducted multiple statistical analyses, we did not adjust for multiple comparisons, as it is not strictly necessary to adjust for multiple comparisons in exploratory studies (49).

## Results

### Baseline Characteristics

Socio-demographic and clinical characteristics of the study participants are presented in **Table 1**. Of the patients, only two had subclinical hypothyroidism (TSH > 5 µU/mL with normal FT4), with a TSH range of 0.63-7.91 µU/mL. These two cases were in the TAU group, which explains significant differences in TSH baseline levels between the two groups (p=0.038). The FT4 concentrations ranged between 0.87 and 1.68 ng/dL and were all within the normal range. There were no significant differences in TSH or FT4 concentrations between men and women (data not shown). The two randomized groups did not differ in socio-demographic characteristics, psychopathology (although a trend towards higher negative symptom scores was found in the TAU group), global functionality, or substance misuse (**Table 1**). In relation to CANTAB cognitive tasks at baseline visit, there were no significant differences with the exception of verbal memory – immediate recall, which was better in the TAU group (30.7 ± 3.1) than in the CRT group (25.8 ± 4.8, p= 0.003).

**Table 1:** Sociodemographic, hormonal, and clinical variables.

	CRT (N=14)	TAU (N=14)	p Value^1^
Female sex	7 (50%)	2 (14.3%)	0.103
Age (years)	26.9 (5.6)	25.4 (4.9)	0.459
Caucasian ethnicity	13 (92.9%)	11 (78.6%)	0.244
Educational level (years of study)	10.3 (2.0)	10.8 (2.6)	0.570
Alcohol use (standard unit/day)	0.7 (1.4)	0.1 (0.1)	0.081
Tobacco (cigarettes/day)	12.6 (6.8)	7.7 (9.5)	0.137
Cannabis Use			0.935
No	5 (35.7%)	5 (35.7%)	
Occasional	5 (35.7%)	6 (42.9%)	
Daily	4 (28.6%)	3 (21.4%)	
PANSS-positive	8 (7-13)	8 (7-14)	0.247
PANSS-negative	9 (10-28)	15 (8-29)	0.406
PANSS-general	26 (21-42)	22 (16-40)	0.087
CDSS	2 (0-15)	2 (0-14)	1.0
PSP	54.6 (15.0)	65.8 (17.8)	0.104
Chlorpromazine equivalent doses (mg/day)	333.4 (179.6)	229.7 (219.0)	0.183
Thyroid hormones			
TSH (µU/mL)	1.86 (0.80)	3.09 (1.90)	0.038
FT4 (ng/dL)	1.20 (0.20)	1.20 (0.21)	0.918

Data represent the mean (SD), median (range) or N (%).

CRT, Cognitive Remediation Therapy; TAU, Treatment as usual; PANSS, Positive and Negative Syndrome Scale; CDSS, Calgary Depression Scale for Schizophrenia; PSP, Personal and Social Performance Scale; TSH, Thyroid Stimulating Hormone; FT4, Free thyroxine.

1 Chi-square test was used for categorical variables. T-test was used for continuous variables. Mann-Whitney U test was used for ordinal variables.

### Wave 1 Analysis (Randomized Phase, CRT vs TAU; Intention-to-Treat).

**Figure 1:**
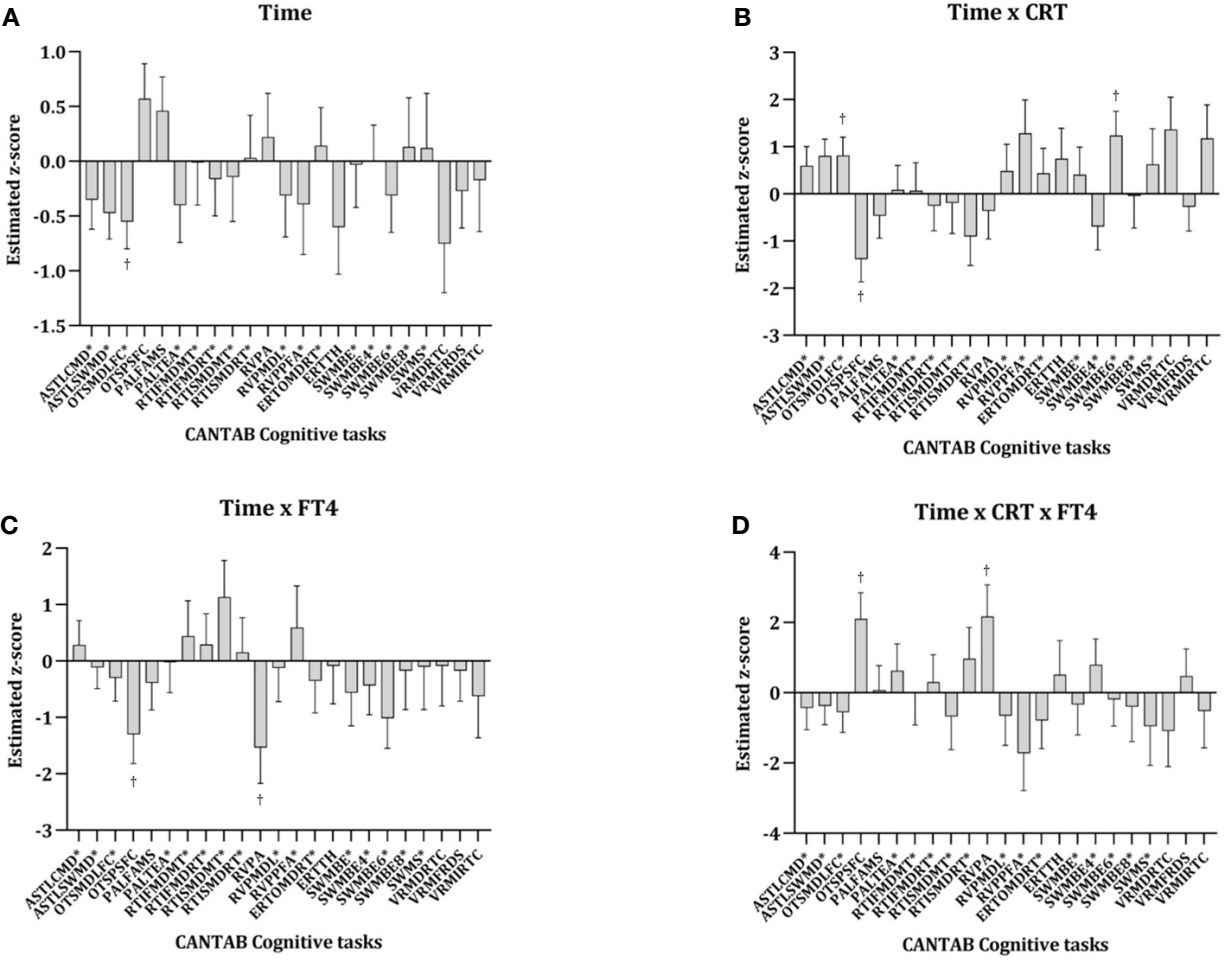
Longitudinal changes in CANTAB cognitive tasks by CRT and FT4 groups: fixed effects dealing with time estimated with linear mixed models. Bars represent the estimated effect and standard error for each cognitive task (z-score) for the following variables and interactions: time **(A)**, time x CRT **(B)**, time x FT4 **(C)** and time x CRT x FT4 **(D)**. The full results of the linear mixed models are also represented in **Table S1** from **Supplementary Material**. CRT, Cognitive remediation therapy; FT4, Free thyroxine. CANTAB Cognitive tasks are described in List S1 from **Supplementary Material**. ^*^Reverse scoring tasks are marked with an asterisk (higher scores representing worse cognitive performance). ^†^p <0.05.

**Figure 2:**
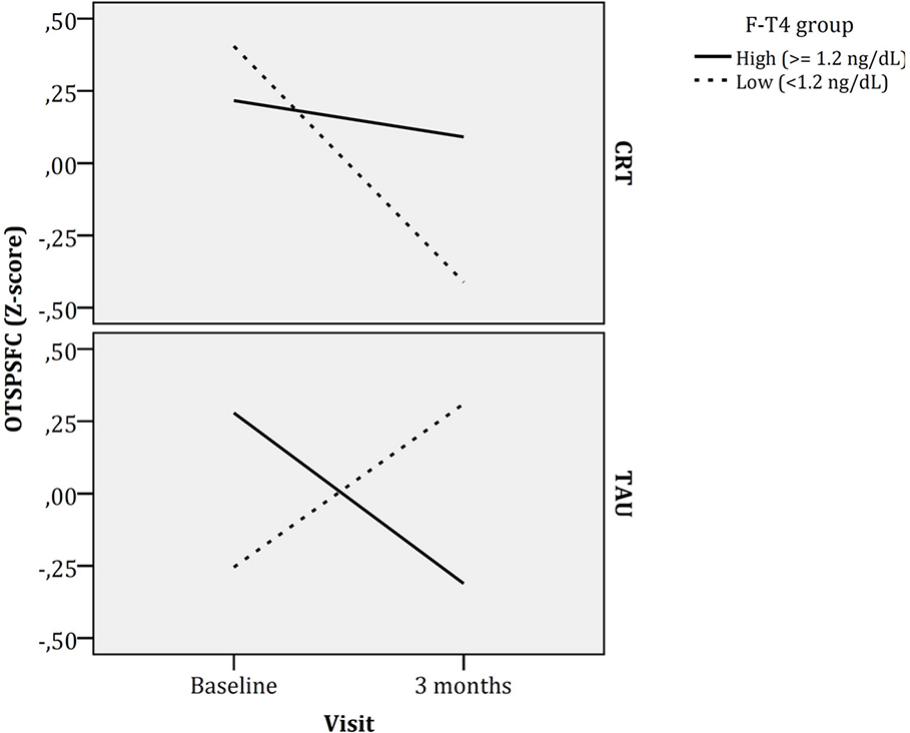
Longitudinal changes in executive function (One Touch Stockings of Cambridge - Problems Solved on First Choice) by CRT and FT4 groups. F-T4, Free thyroxin; CRT, Cognitive remediation therapy; TA5U, Treatment as usual.

Longitudinal cognitive changes by treatment arm are described in **Table 2**. The estimated fixed effects of factors and covariates and interactions calculated with the linear mixed models are reported in **Table S1** from the **Supplementary Material**. These effects, that considered z-scores for each CANTAB cognitive variable, have been also described in **Figure 1**. For easing the interpretation of these effects, those cognitive variables that show an inverse relationship with cognitive performance (higher scores reflecting worse cognitive performance) have been indicated with an asterisk. Therefore, positive estimated fixed effects (z-scores for each cognitive task) would represent cognitive improvement over time in positive cognitive variables but cognitive worsening over time in inverse cognitive variables (marked with an asterisk). As it can be seen in **Figure 1**, longitudinal changes in OTS Problems Solved on First Choice (OTSPSFC), a measure of executive functioning, differed between treatment (TAU vs CRT) and FT4 (low vs high) groups, as the interaction between time x CRT x FT4 group was significant. This triple interaction is also represented in **Figure 2**, showing that patients with higher FT4 levels worsened in this measure in both CRT and TAU groups whereas patients with lower FT4 levels had a different pattern depending on the treatment arm (worsening when receiving CRT and improvement when receiving TAU).

**Table 2:** Longitudinal changes in cognitive measures (raw scores) by treatment arm.

CANTAB task^1^	CANTAB variable^2^	TAU (n=14)		CRT (n=14)	
		Pre	Post	Pre	Post
AST	ASTLCMD*	600.7 (135.7)	565.3 (147.6)	635.2 (157.7)	642.5 (261.7)
	ASTLSWMD*	793.8 (185)	681.0 (199.3)	750.4 (205.3)	749.3 (231.6)
OTS	OTSMDLFC*	11649.0 (4637.1)	7694.8 (3398.8)	12753.4 (5709.1)	11387.9 (7419.0)
	OTSPSFC	9.4 (2.1)	9.5 (2.4)	9.9(2.7)	8.8 (3.5)
PAL	PALFAMS	14.3 (5.5)	15.9 (2.8)	11.1 (3.5)	11.0 (3.6)
	PALTEA*	12.4 (16.3)	6.0 (6.4)	18.9 (12.2)	17.3 (10.1)
RTI	RTIFMDMT*	266.4 (68.3)	281.7 (98.6)	287.7 (87.1)	297.9 (132.6)
	RTIFMDRT*	403.2 (65.3)	398.3 (42.9)	438.4 (67.2)	431.3 (72.4)
	RTISMDMT*	227.5 (65.7)	248.8 (113.3)	237.4 (81.5)	226.9 (64.5)
	RTISMDRT*	348.9 (58.8)	353.1 (38.4)	381.8 (87.0)	367.5 (66.2)
RVP	RVPA	0.84 (0.04)	0.82 (0.08)	0.83 (0.03)	0.84 (0.07)
	RVPMDL*	509.6 (109.5)	460.7 (83.7)	548.0 (127.2)	494.4 (135.7)
	RVPPFA*	0.026 (0.057)	0.014 (0.018)	0.036 (0.045)	0.055 (0.118)
ERT	ERTOMDRT*	1499.9 (453.2)	1356.9 (500.4)	1939.2 (651.3)	1867.8 (1534.4)
	ERTTH	29.14 (4.28)	26.25 (7.58)	26 (4.10)	27.54 (3.41)
SWM	SWMBE*	15.2 (8.0)	12.7 (8.1)	15.5 (19.8)	14.5 (7.9)
	SWMBE4*	1.1 (1.4)	0.8 (1.3)	1.4 (1.2)	0.8 (0.9)
	SWMBE6*	5.5 (3.6)	3.1 (2.1)	4.1 (3.5)	4.9 (3.5)
	SWMBE8*	8.5 (5.7)	8.8 (5.8)	10.2 (6.3)	9.1 (4.9)
	SWMS*	8.46 (2.8)	8.6 (2.5)	8.2 (2.4)	8.9 (2.3)
VRM	VRMDRTC	30.6 (4.5)	27.8 (5.7)	27.4 (3.7)	27.4 (3.7)
	VRMFRDS	6.7 (3.4)	6.0 (2.5)	4.9 (2.1)	3.8 (1.8)
	VRMIRTC	30.7 (3.1)	28.9 (4.4)	25.8 (4.8)	26.6 (4.3)

TAU, Treatment as usual; CRT, Cognitive remediation therapy; AST, Attention Switching Task (assessing attention and response latencies); OTS, One Touch Stockings of Cambridge (assessing executive functioning: spatial planning and working memory); PAL, Paired Associates Learning (assessing visual memory and new learning); RTI, Reaction Time (assessing reaction time: motor and mental response speeds, as well as measures of movement time, reaction time, response accuracy and impulsivity); RVP, Rapid Vision Information Processing (assessing sustained attention); ERT, Emotion Recognition Task (assessing theory of mind processes); SWM, Spatial Working Memory (assessing Spatial Working Memory: retention and manipulation of visuospatial information, strategy and working memory errors); VRM, Verbal Recognition Memory (assessing verbal memory and new learning).

^1^The definition of all CANTAB tasks (and CANTAB variables) is described in List S1 from **Supplementary Material**.

^2^For all cognitive tasks, lower scores reflect better cognitive performance unlike OTSPSFC, PALFAMS, RVPA, ERTTH, and all VRM tasks (which are the opposite; higher scores reflect better cognitive performance). Reverse scores have been marked with an asterisk (*), indicating that higher scores reflect worse cognitive performance.

**Figure 3:**
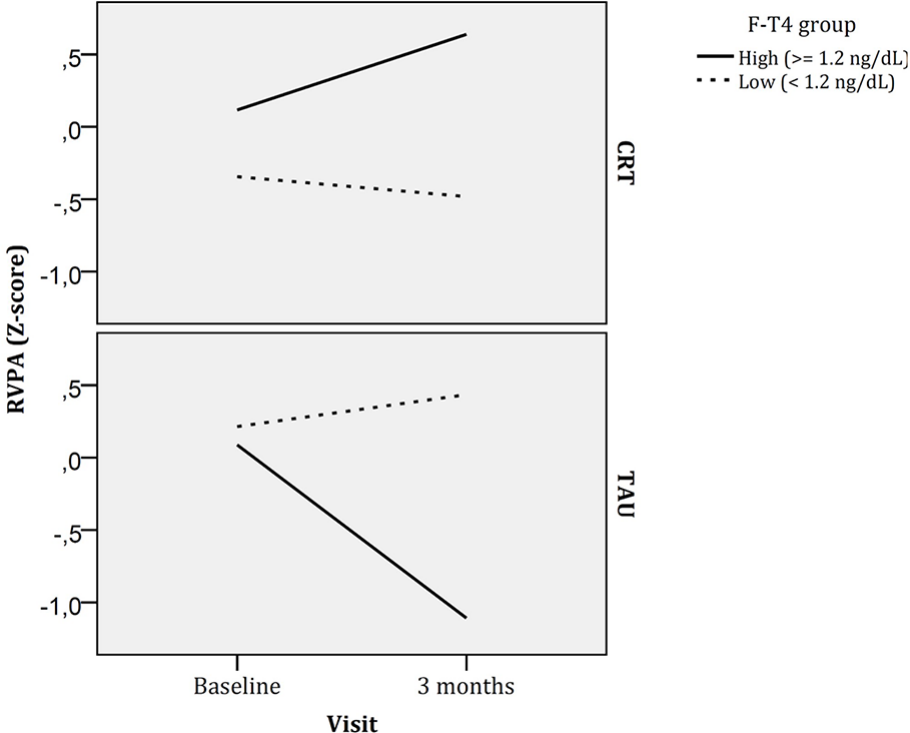
Longitudinal changes in rapid visual information processing by CRT and FT4 groups. F-T4, Free thyroxin; CRT, Cognitive remediation therapy; TAU, Treatment as usual.

Another significant triple interaction (time x CRT x FT4 group) was found for rapid visual information processing (RVPA, a measure that reflects how good a subject is for detecting target sequences). Patients with higher FT4 concentrations in the TAU arm worsened in this measure (**Figure 3**), but improved if were receiving CRT. In contrast, patients with lower FT4 concentrations showed less cognitive changes in this cognitive task over time independently of their treatment arm (CRT or TAU).

As it can be seen in **Table S1** and **Figure 1**, CRT was not associated with significant changes over time in most cognitive variables (most time x CRT interactions were non-significant), and ever worsened in a two cognitive tests dealing with executive functioning (OTS) and spatial working memory (SWMBE6, a measure of working memory errors; this task has also notable executive function demands).

### Wave 2 Analyses (Pre-Post Changes After CRT in the Patients Completing One Third of the Sessions; Per-Protocol Analysis).

In this secondary analysis, we included all patients assigned to CRT who completed one third of the sessions (N= 14 participants). There were no significant differences in CANTAB cognitive tasks between patients who did not engage in the CRT and those who completed at least one third of the sessions (data not shown). CRT treatment was not associated with cognitive improvement in any task (**Table 3**), as the time effect was non-significant for all measures but the SWMBE6 (patients worsened in this task after CRT). A time x FT4 interaction was found for two cognitive tasks dealing with spatial working memory (SWMBE6) and verbal memory (VRMDRTC, delayed recognition of words). These interactions are shown in **Figures S2** and **S3** from the **Supplementary Material**. As it can be seen in **Figure S2**, patients with lower FT4 concentrations made more errors in the spatial working memory task involving 6 tokens after CRT whereas patients with higher FT4 concentrations improved over time in this task after CRT. In relation to delayed verbal memory, those patients with higher FT4 concentrations worsened in this task whereas those patients with lower FT4 concentrations improved after CRT (**Figure S3**).

**Table 3:** Results of the linear mixed models exploring longitudinal changes in cognition in the patients receiving at least one third of sessions of cognitive remediation.

CANTAB task	CANTAB variable^2^	CRT (n=14)		Estimated fixed effects (calculated with z-scores)							
				Time		FT4 group		TSH		Time x FT4 group	
		Pre	Post	Effect (SE)	P	Effect (SE)	P	Effect (SE)	P	Effect (SE)	P
AST	ASTLCMD*	624.9 (158.2)	677.7 (261.2)	0.05 (0.34)	0.895	-0.01 (0.44)	0.989	0.11 (0.22)	0.638	0.01 (0.50)	0.977
	ASTLSWMD*	714.1 (177.2)	720.6 (231.3)	0.13 (0.30)	0.673	0.31 (0.51)	0.550	0.34 (0.25)	0.897	-0.22 (0.45)	0.631
OTS	OTSMDLFC*	11301.1 (6648.3)	11241.5 (7298.9)	0.15 (0.20)	0.466	0.12 (0.57)	0.838	-0.04 (0.28)	0.887	-0.38 (0.31)	0.247
	OTSPSFC	9.21 (3.0)	9.50 (3.8)	-0.19 (0.32)	0.562	-0.02 (0.52)	0.969	0.47 (0.26)	0.859	0.64 (0.48)	0.209
PAL	PALFAMS	12.5 (4.9)	12.4 (3.8)	-0.12 (0.41)	0.784	0.14 (0.54)	0.796	**0.48 (0.19)**	**0.028**	0.23 (0.63)	0.720
	PALTEA*	16.6 (14.7)	15.0 (11.2)	-0.30 (0.37)	0.431	-0.62 (0.53)	0.267	**-0.49 (0.20)**	**0.032**	0.42 (0.57)	0.474
RTI	RTIFMDMT*	251.9 (68.0)	275.9 (76.8)	0.03 (0.40)	0.941	-0.08 (0.54)	0.886	0.14 (0.24)	0.562	0.70 (0.61)	0.272
	RTIFMDRT*	401.3 (65.1)	414.1 (66.8)	0.71 (0.34)	0.840	0.09 (0.57)	0.876	-0.19 (0.26)	0.469	0.30 (0.53)	0.583
	RTISMDMT*	227.7 (67.4)	243.0 (78.1)	0.06 (0.36)	0.864	-0.10 (0.53)	0.862	0.20 (0.25)	0.435	0.35 (0.55)	0.536
	RTISMDRT*	357.5 (57.5)	363.1 (65.5)	-0.04 (0.36)	0.903	-0.07 (0.55)	0.894	-0.14 (0.26)	0.591	0.32 (0.55)	0.571
RVP	RVPA	0.83 (0.03)	0.86 (0.03)	0.39 (0.27)	0.176	0.22 (0.52)	0.680	0.08 (0.23)	0.721	0.79 (0.41)	0.080
	RVPMDL*	535.3 (103.7)	496.0 (113.7)	-0.35 (0.48)	0.476	-0.52 (0.51)	0.329	-0.37 (0.18)	0.068	-0.02 (0.73)	0.978
	RVPPFA*	0.043 (0.067)	0.020 (0.018)	-0.49 (0.44)	0.285	-0.35 (0.74)	0.652	-0.09 (0.09)	0.371	0.09 (0.70)	0.897
ERT	ERTOMDRT*	1801.4 (614.0)	1855.1 (1462.2)	0.32 (0.38)	0.428	-0.09 (0.31)	0.766	-0.19 (0.15)	0.242	-0.62 (0.59)	0.311
	ERTTH	28.4 (4.8)	28.2 (3.2)	-0.09 (0.37)	0.806	-0.14 (0.64)	0.836	0.19 (0.23)	0.433	0.13 (0.57)	0.817
SWM	SWMBE*	12.8 (9.6)	14.2 (8.9)	0.53 (0.34)	0.152	0.004 (0.52)	0.994	-0.38 (0.25)	0.155	-0.70 (0.53)	0.215
	SWMBE4*	0.93 (1.33)	0.93 (1.0)	0.33 (0.37)	0.395	0.61 (0.63)	0.354	-0.06 (0.24)	0.813	-0.76 (0.56)	0.203
	SWMBE6*	3.4 (3.4)	4.2 (3.4)	**0.82 (0.23)**	**0.004**	0.40 (0.53)	0.471	-0.23 (0.25)	0.384	**-1.31 (0.35)**	**0.003**
	SWMBE8*	8.5 (6.2)	9.3 (5.5)	0.27 (0.45)	0.554	-0.33 (0.52)	0.539	-0.34 (0.23)	0.169	-0.19 (0.69)	0.790
	SWMS*	7.5 (2.7)	9.0 (1.8)	0.91 (0.45)	0.067	0.12 (0.65)	0.854	0.02 (0.19)	0.903	-0.68 (0.69)	0.349
VRM	VRMDRTC	28.3 (4.8)	28.4 (4.8)	-0.71 (0.37)	0.084	**1.69 (0.32)**	**< 0.001**	0.09 (0.16)	0.589	**-1.69 (0.55)**	**0.010**
	VRMFRDS	5.5 (2.7)	4.3 (1.9)	-0.69 (0.46)	0.164	-0.09 (0.68)	0.894	0.32 (0.18)	0.113	0.41 (0.71)	0.577
	VRMIRTC	27.0 (5.2)	27.9 (3.6)	0.69 (0.57)	0.250	1.07 (0.60)	0.099	0.03 (0.15)	0.870	-1.14 (0.87)	0.211

CRT, Cognitive remediation therapy; TSH, Thyroid-stimulating hormone; FT4, Free thyroxine; AST, Attention Switching Task; OTS, One Touch Stockings of Cambridge; PAL, Paired Associates Learning; RTI, Reaction Time; RVP, Rapid Vision Information Processing; ERT, Emotion Recognition Task; SWM, Spatial Working Memory; VRM, Verbal Recognition Memory.

1 The definition of all CANTAB tasks is described in List S1 from **Supplementary Material**.

2 For all cognitive tasks, lower scores reflect better cognitive performance unlike OTSPSFC, PALFAMS, RVPA, ERTTH, and all VRM tasks (which are the opposite; higher scores reflect better cognitive performance). Reverse scores have been marked with an asterisk (*), indicating that higher scores reflect worse cognitive performance.

All statistically significant domains (p < 0.05) have been highlighted in bold text.

## Discussion

The present study highlights the role of hypothalamic–pituitary–thyroid axis hormones, particularly FT4, in cognitive changes over time in a sample of young patients with early psychosis who received CRT, especially in sustained attention and executive functions. In the secondary analysis, including only the patients who completed at least on third of the CRT, FT4 appeared to moderate the response to the CRT in relation to spatial working memory and delayed verbal memory.

Our clinical trial was designed as a pilot study to explore whether thyroid hormones could predict the clinical response to CRT. We included a sample of ROP patients because we were interested in exploring the potential pro-cognitive effect of CRT in early stages of the illness, and we also aimed to identify biomarkers that would help to determine which patients would benefit from an early intervention. When controlling for TSH and FT4 concentrations, we failed to detect a specific effect of the intervention (CRT) compared with TAU. This could be explained by several reasons such as a low proportion of participation in sessions or the small sample size, which could imply a lack of statistical power for detecting small effects. Our sample comprised ROP patients; this could also explain why both groups (CRT and TAU) improved over time because ROP patients are known to improve in cognitive abilities in the first years of the illness (50, 51). Moreover, several patients became enrolled in educational or job-seeking activities, which could also contribute to improved cognition over time.

Previous studies have reported that subtle deficits in working memory and executive function likely exist in subclinical thyroid disease hypothesizing that the degree of cognitive dysfunction is probably related to the degree of thyroid dysfunction and subclinical thyroid disease (34). Several studies conducted in psychiatric populations have reported poorer cognitive performance (in processing speed and verbal memory) in patients with bipolar disorder and subclinical hypothyroidism, as well as cognitive improvements with T3 administration in patients receiving lithium or electroconvulsive therapy (32). In our study, patients had FT4 levels within the normal range, which is in accordance with previous results from our group (37) that noted that subtle differences in FT4 levels may be important in cognitive performance in people with early psychosis. The cited cross-sectional study found that increased FT4 levels were associated with better performance in the attention/vigilance domain, especially in subjects with affective psychosis. In another longitudinal study from our group (38), both higher and lower FT4 concentrations (although within normal limits) were associated with poorer cognitive outcome in attention/vigilance one year later.

As the sample size of our study was small, we decided to compare high vs low FT4 concentrations using the median of our sample (1.2 ng/dL). For this reason, we could not explore a “U-shaped” pattern as in our previous study (38). In our current study, higher FT4 levels moderated longitudinal changes in two cognitive tasks that imply attention (sustained attention and immediate verbal learning), although the direction differed depending upon the cognitive task: improved sustained attention and spatial working memory after CRT, whereas worsened delayed verbal memory recognition. Although this finding might seem contradictory with our previous longitudinal study (38) that reported worsening in attention and vigilance in those patients with higher FT4, it is important to underscore that cut-offs for groups differ between both studies (1.54 ng/dL vs 1.2 ng/dL). In our current study, only two patients had a FT4 concentration above 1.54 ng/dL. This fact, altogether with the finding in the study by Labad et al. (38), that reported improvements in attention in those patients with FT4 levels between 1.16 and 1.54 ng/dL, makes less contradictory both results. It does suggest, however, that subtle deviations in FT4 concentrations might moderate the cognitive response to a CRT in some cognitive domains. As our study is the first of its kind in people with ROP, larger studies are needed to replicate our findings and to also explore whether a potential “U shape” pattern does also apply for the cognitive response to a CRT.

Although several classic studies have found contradictory results between cross-sectional associations among TSH, FT4, FT3, and cognitive performance (39, 52), there are no studies exploring the potential role of thyroid hormones as a predictor of response to CRT. Our study needs to be considered a pilot clinical trial and replicated before drawing definitive conclusions. The secondary analysis conducted in the subsample with a greater attendance to CRT sessions shows promising results, suggesting the potential moderating effects of thyroid hormones on cognitive changes. If these results are confirmed in future studies with larger samples, a tailored therapy approach taking into account specific biomarkers (e.g., FT4) to maximize its potential benefits is suggested.

The physiopathology connecting thyroid hormones and cognition is not fully known to date. It is known that thyroid hormones have an association with the polymerization of astrocytes. Those cells may contribute to behavioral disorders related to reward circuits, and reactive astrogliosis is a well-known feature of Alzheimer’s disease (53). Several studies have detected a decreased number of astrocytes in cortical and subcortical areas in patients with schizophrenia (54). Moreover, dystrophic and swollen astrocytes have been found in psychotic patients, and those alterations progressed with the duration of the illness (55). Recently, O’Donovan et al. hypothesized that increased adenosine kinase activity in astrocytes may reduce extracellular adenosine, with subsequent hypofunction on the adenosine neurotransmitter system, which is altered in schizophrenia (56). Antipsychotic treatment is another factor that could also contribute to the reduction of astrocytes because astrocytes express dopamine receptors. It could also be that antipsychotic treatment reduces the number of astrocytes, as astrocytes express dopamine receptors, and animal studies have demonstrated a lower astrocyte number in macaque monkeys chronically exposed to antipsychotics (57). Thus, it seems that thyroid hormones could intervene in cognitive changes through their actions on astrocytes, affecting the morphology, functioning, and number of these cells in psychotic patients.

Some limitations of our study need to be addressed. Of the participants in our study, a half of the sample did not engage with at least one third of the sessions of CRT. Nevertheless, these rates are similar to those in previously published studies from other groups (58). This fact may be influenced by the study design because we included a randomized phase and half of the patients received TAU. Several patients failed to complete the CRT because they enrolled in educational or job-seeking activities. The low proportion of completed CRT sessions could make the detection of a positive effect of CRT on cognitive changes over time difficult, as the first analyses (global sample, randomized phase, intention-to-treat analysis) included all patients independently of the proportion of attended sessions. We decided to define the attendance at one third of the sessions as a significant treatment because previous studies suggest that even short interventions (5–15 h) could be associated with mild cognitive improvements (15). Another limitation of our study was not including the FT3 concentrations, as previous studies suggest that FT3 levels are associated with better cognitive function in schizophrenia patients (36). Finally, one of the most important limitations was the small sample size, which does not allow for conducting sub-analyses by diagnoses or by sex, due to a lack of statistical power. As the sample size of our study was small, we preferred not to adjust for potential covariables (e.g. negative symptoms, tobacco, or cannabis use) for avoiding the instability of the model.

In summary, FT4 concentrations moderate longitudinal cognitive changes over time in ROP patients, mainly in specific domains such as sustained attention, executive functions, and verbal and working memory. It appears that FT4 concentrations may be able to predict the response to CRT in people with early psychosis; however, the pilot nature of our study makes it necessary to replicate our findings in future studies.

## Data Availability Statement

The original contributions presented in the study are included in the article/**Supplementary Material**; further inquiries can be directed to the corresponding author.

## Ethics Statement

The study was reviewed and approved by the Drug Research Ethics Committee (CEIm) Parc Taulí. All patients provided their written informed consent to participate in this study.

## Author Contributions

JL designed the study, wrote the protocol, and performed the statistical analysis. FE wrote the first draft of the manuscript, which was supervised by JL. FE, AG, JC, SP-M, and MA evaluated the cognitive and clinical function. WZ and JC conducted the CR intervention. RA and LL participated in the collection and processing of biological samples. JB, MT, JL, and IM participated in the recruitment. All authors contributed to the article and approved the submitted version.

## Funding

This study was supported by grants from the Carlos III Health Institute through the Ministry of Economy and Competitiveness (PI15/01386) and the European Regional Development Fund (ERDF) “A way to build Europe”. JL and IM received an Intensification of the Research Activity Grant (SLT006/17/00012; SLT008/18/00074) from the Health Department of the Generalitat de Catalunya.

## Conflict of Interest

The authors declare that the research was conducted in the absence of any commercial or financial relationships that could be construed as a potential conflict of interest.

## References

[1] Green MF Kern RS Heaton RK Longitudinal studies of cognition and functional outcome in schizophrenia: Implications for MATRICS. Schizophr Res (2004) 72(1):41–51. 10.1016/j.schres.2004.09.009 15531406

[2] Leeson VC Harrison I Ron MA Barnes TRE Joyce EM The effect of cannabis use and cognitive reserve on age at onset and psychosis outcomes in first-episode schizophrenia. Schizophr Bull (2012) 38(4):873–80. 10.1093/schbul/sbq153 PMC3406524 21389110

[3] Santesteban-Echarri O Paino M Rice S González-Blanch C McGorry P Gleeson J Predictors of functional recovery in first-episode psychosis: A systematic review and meta-analysis of longitudinal studies. Clin Psychol Rev [Internet] (2017) 58:59–75. 10.1016/j.cpr.2017.09.007 29042139

[4] Opler LA Medalia A Opler MG Stahl SM Pharmacotherapy of cognitive deficits in schizophrenia. CNS Spectr (2014) 19(2):142–56. 10.1017/S1092852913000771 24229725

[5] Wykes T A Meta-Analysis of Cognitive Remediation for Schizophrenia: Methodology and Effect Sizes. Am J Psychiatry (2011) 168:472–85. 10.1176/appi.ajp.2010.10060855 21406461

[6] McGurk SR Twamley EW Sitzer DI McHugo GJ Mueser KT A meta-analysis of cognitive remediation in schizophrenia. Am J Psychiatry (2007) 164(12):1791–802. 10.1176/appi.ajp.2007.07060906 PMC3634703 18056233

[7] Lee RSC Redoblado-Hodge MA Naismith SL Hermens DF Porter MA Hickie IB Cognitive remediation improves memory and psychosocial functioning in first-episode psychiatric out-patients. Psychol Med (2013) 43(6):1161–73. 10.1017/S0033291712002127 PMC3642720 23237010

[8] Lewandowski KE Cognitive Remediation for the Treatment of Cognitive Dysfunction in the Early Course of Psychosis. Harv Rev Psychiatry (2016) 24(2):164–72. 10.1097/HRP.0000000000000108 26954599

[9] Barlati S De Peri L Deste G Fusar-Poli P Vita A Cognitive Remediation in the Early Course of Schizophrenia: A Critical Review. Curr Pharm Des (2012) 18(4):534–41. 10.2174/138161212799316091 22239585

[10] Bell MD Bryson G Work Rehabilitation in Schizophrenia: Does Cognitive Impairment Limit Improvement? Schizophr Bull (2001) 27(2):269–79. 10.1093/oxfordjournals.schbul.a006873 11354594

[11] Cella M Preti A Edwards C Dow T Wykes T Cognitive remediation for negative symptoms of schizophrenia: A network meta-analysis. Clin Psychol Rev (2017) 52:43–51. 10.1016/j.cpr.2016.11.009 27930934

[12] Ventura J Subotnik KL Gretchen-Doorly D Casaus L Boucher M Medalia A Cognitive remediation can improve negative symptoms and social functioning in first-episode schizophrenia: A randomized controlled trial. Schizophr Res (2019) 203:24–31. 10.1016/j.schres.2017.10.005 29128326 PMC6589092

[13] Wykes T Huddy V Cognitive remediation for schizophrenia: It is even more complicated. Curr Opin Psychiatry (2009) 22(2):161–7. 10.1097/YCO.0b013e328322fbf4 19553870

[14] Grynszpan O Perbal S Pelissolo A Fossati P Jouvent R Dubal S Efficacy and specificity of computer-assisted cognitive remediation in schizophrenia: A meta-analytical study. Psychol Med (2011) 41(1):163–73. 10.1017/S0033291710000607 20380784

[15] Morin L Franck N Rehabilitation interventions to promote recovery from schizophrenia: A systematic review. Front Psychiatry (2017) 8:100. 10.3389/fpsyt.2017.00100 28659832 PMC5467004

[16] Murthy NV Mahncke H Wexler BE Maruff P Inamdar A Zucchetto M Computerized cognitive remediation training for schizophrenia: An open label, multi-site, multinational methodology study. Schizophr Res (2012) 139(1–3):87–91. 10.1016/j.schres.2012.01.042 22342330

[17] Reser MP Slikboer R Rossell SL A systematic review of factors that influence the efficacy of cognitive remediation therapy in schizophrenia. Aust N Z J Psychiatry (2019) 53(7):624–41. 10.1177/0004867419853348 31177813

[18] McGurk SR Mueser KT Response to cognitive rehabilitation in older versus younger persons with severe mental illness. Am J Psychiatr Rehab (2008) 11(1):90–105. 10.1080/15487760701853136

[19] Wykes T Reeder C Landau S Matthiasson P Haworth E Hutchinson C Does age matter? Effects of cognitive rehabilitation across the age span. Schizophr Res (2009) 113(2–3):252–8. 10.1016/j.schres.2009.05.025 19524409

[20] Pillet B Morvan Y Todd A Franck N Duboc C Grosz A Cognitive remediation therapy (CRT) benefits more to patients with schizophrenia with low initial memory performances. Disabil Rehabil (2015) 37(10):846–53. 10.3109/09638288.2014.946153 25109501

[21] Seccomandi B Tsapekos D Newbery K Wykes T Cella M A systematic review of moderators of cognitive remediation response for people with schizophrenia. Schizophr Res Cogn (2019) 19:100160. 10.1016/j.scog.2019.100160 31828023 PMC6889639

[22] Bosia M Bechi M Pirovano A Buonocore M Lorenzi C Cocchi F COMT and 5-HT1A-receptor genotypes potentially affect executive functions improvement after cognitive remediation in schizophrenia. Heal Psychol Behav Med (2014) 2(1):509–16. 10.1080/21642850.2014.905206 PMC4346068 25750798

[23] Lindenmayer JP Kahn A Lachman H McGurk SR Goldring A Thanju A COMT genotype and response to cognitive remediation in schizophrenia. Schizophr Res (2015) 168(1–2):279–84. 10.1016/j.schres.2015.07.037 PMC4591188 26255563

[24] Bosia M Zanoletti A Spangaro M Buonocore M Bechi M Cocchi F Factors affecting cognitive remediation response in schizophrenia: The role of COMT gene and antipsychotic treatment. Psychiatry Res (2014) 217(1–2):9–14. 10.1016/j.psychres.2014.02.015 24656901

[25] Fisher M Mellon SH Wolkowitz O Vinogradov S Neuroscience-informed auditory training in schizophrenia: A final report of the effects on cognition and serum brain-derived neurotrophic factor. Schizophr Res Cognit (2016) 3:1–7. 10.1016/j.scog.2015.10.006 26705516 PMC4685735

[26] Penadés R López-Vílchez I Catalán R Arias B González-Rodríguez A García-Rizo C BDNF as a marker of response to cognitive remediation in patients with schizophrenia: A randomized and controlled trial. Schizophr Res (2018) 197(2018):458–64. 10.1016/j.schres.2017.12.002 29274733

[27] Spangaro M Bosia M Bechi M Buonocore M Cocchi F Guglielmino C Neurobiology of cognitive remediation in schizophrenia: Effects of EAAT2 polymorphism. Schizophr Res (2018) 202(2018):106–10. 10.1016/j.schres.2018.06.059 30539765

[28] Keshavan MS Eack SM Wojtalik JA Prasad KMR Francis AN Bhojraj TS A broad cortical reserve accelerates response to cognitive enhancement therapy in early course schizophrenia. Schizophr Res (2011) 130(1–3):123–9. 10.1016/j.schres.2011.05.001 PMC3209759 21645997

[29] Penadés R Pujol N Catalán R Masana G García-Rizo C Bargalló N Cortical thickness in regions of frontal and temporal lobes is associated with responsiveness to cognitive remediation therapy in schizophrenia. Schizophr Res (2016) 171(1–3):110–6. 10.1016/j.schres.2016.01.006 26777884

[30] Subramaniam K Gill J Fisher M Mukherjee P Nagarajan S Vinogradov S White matter microstructure predicts cognitive training-induced improvements in attention and executive functioning in schizophrenia. Schizophr Res (2018) 193:276–83. 10.1016/j.schres.2017.06.062 PMC5999406 28689758

[31] Penadés R Bosia M Catalán R Spangaro M García-Rizo C Amoretti S The role of genetics in cognitive remediation in schizophrenia: A systematic review. Schizophr Res Cogn (2019) 19:100146. 10.1016/j.scog.2019.100146 31832337 PMC6889757

[32] Tost M Monreal JA Armario A Barbero JD Cobo J García-Rizo C Targeting Hormones for Improving Cognition in Major Mood Disorders and Schizophrenia: Thyroid Hormones and Prolactin. Clin Drug Investig (2019) 40(1):1–14. 10.1007/s40261-019-00854-w 31612424

[33] Leonard JL Nongenomic Actions of Thyroid Hormone in Brain Development. Steroids (2008) 73(9–10):1008–12. 10.1016/j.steroids.2007.12.016 PMC2601565 18280526

[34] Samuels MH Thyroid disease and cognition. Endocrinol Metab Clin North Am (2014) 43(2):529–43. 10.1016/j.ecl.2014.02.006 24891176

[35] Othman SS Kadir KA Hassan J Hong GK Singh BB Raman N High prevalence of thyroid function test abnormalities in chronic schizophrenia. Aust N Z J Psychiatry (1994) 28(4):620–4. 10.3109/00048679409080785 7794205

[36] Ichioka S Terao T Hoaki N Matsushita T Hoaki T Triiodothyronine may be possibly associated with better cognitive function and less extrapyramidal symptoms in chronic schizophrenia. Prog Neuropsychopharmacol Biol Psychiatry (2012) 39(1):170–4. 10.1016/j.pnpbp.2012.06.008 22750309

[37] Barbero JD Gutiérrez-Zotes A Montalvo I Creus M Cabezas Á Solé M Free thyroxine levels are associated with cognitive abilities in subjects with early psychosis. Schizophr Res (2014) 166(1–3):37–42. 10.1016/j.schres.2015.04.030 25982813

[38] Labad J Barbero JD Gutiérrez-Zotes A Montalvo I Creus M Cabezas Free thyroxine levels are associated with cognitive changes in individuals with a first episode of psychosis: A prospective 1-year follow-up study. Schizophr Res (2016) 171(1–3):182–6. 10.1016/j.schres.2016.01.036 26805411

[39] Ewins DL Rossor MN Butler J Rogues PK Mullen MJ McGregor AM Association between autoimmune thyroid disease and Familial Alzheimers disease. Clin Endocrinol (Oxf) (1991) 35(1):93–6. 10.1111/j.1365-2265.1991.tb03502.x 1889144

[40] Fernandez-Gonzalo S Turon M Jodar M Pousa E Hernandez-Rambla C García R A new computerized cognitive and social cognition training specifically designed for patients with schizophrenia/schizoaffective disorder in early stages of illness: A pilot study. Psychiatry Res (2015) 228(3):501–9. 10.1016/j.psychres.2015.06.007. 26163731

[41] Caballero-Hernández R Vila-Forcen A Fernandez-Gonzalo S Martínez-Moreno JM Turon M Sánchez-Carrión R (2014). “Video-Based Tasks for Emotional Processing Rehabilitation in Schizophrenia,” in XIII Mediterranean Conference on Medical and Biological Engineering and Computing 2013 IFMBE Proceedings., Vol. 41 1779–82.

[42] Kay S Fiszbein A Vital-Herne M Fuentes L The Positive and Negative Syndrome Scale-Spanish Adaptation. J Nerv Ment Dis (1990) 178(8):510–7. 10.1097/00005053-199008000-00007 2380697

[43] Addington D Addington J Schissel B A depression rating scale for schizophrenics. Schizophr Res (1990) 3(4):247–51. 10.1016/0920-9964(90)90005-R 2278986

[44] Garcia-Portilla MP Saiz PA Bousoño M Bascaran MT Guzmán-Quilo C Bobes J Validación de la versión española de la escala de Funcionamiento Personal y Social en pacientes ambulatorios con esquizofrenia estable o inestable. Rev Psiquiatr Salud Ment (2011) 4(1):9–18. 10.1016/j.rpsm.2010.11.003 23446097

[45] Gardner DM Murphy AL O’Donnell H Centorrino F Baldessarini RJ International consensus study of antipsychotic dosing. Am J Psychiatry (2010) 167:686–93. 10.1176/appi.ajp.2009.09060802 20360319

[46] Sahakian BJ Owen AM Computerized assessment in neuropsychiatry using CANTAB: Discussion paper. J R Soc Med (1992) 85(7):399–402. 1629849 PMC1293547

[47] Julious SA Sample size of 12 per group rule of thumb for a pilot study. Pharmaceut Statist (2005) 4:287–91. 10.1002/pst.185

[48] Whitehead AL Julious SA Cooper CL Campbell MJ Estimating the sample size for a pilot randomised trial to minimise the overall trial sample size for the external pilot and main trial for a continuous outcome variable. Stat Methods Med Res (2016) 25(3):1057–73. 10.1177/0962280215588241 PMC4876429 26092476

[49] Bender R Lange S Adjusting for multiple testing-when and how? J Clin Epidemiol (2001) 54(4):343–9. 10.1016/s0895-4356(00)00314-0 11297884

[50] Olivier MR Killian S Chiliza B Asmal L Schoeman R Oosthuizen PP Cognitive performance during the first year of treatment in first-episode schizophrenia: A case-control study. Psychol Med (2015) 45(13):2873–83. 10.1017/S0033291715000860 25998030

[51] Bora E Murray RM Meta-analysis of cognitive deficits in ultra-high risk to psychosis and first-episode psychosis: Do the cognitive deficits progress over, or after, the onset of psychosis? Schizophr Bull (2014) 40(4):744–55. 10.1093/schbul/sbt085 PMC4059428 23770934

[52] Wahlin Å Wahlin TBR Small BJ Bäckman L Influences of thyroid stimulating hormone on cognitive functioning in very old age. J Gerontol B Psychol Sci Soc Sci (1998) 53(4):234–9. 10.1093/geronb/53B.4.P234 9679515

[53] Sofroniew MV Vinters HV Astrocytes: Biology and pathology. Acta Neuropathol (2010) 119(1):7–35. 10.1007/s00401-009-0619-8 20012068 PMC2799634

[54] Williams MR Hampton T Pearce RKB Hirsch SR Ansorge O Thom M Astrocyte decrease in the subgenual cingulate and callosal genu in schizophrenia. Eur Arch Psychiatry Clin Neurosci (2013) 263(1):41–52. 10.1007/s00406-012-0328-5 22660922

[55] Kolomeets NS Uranova N Ultrastructural abnormalities of astrocytes in the hippocampus in schizophrenia and duration of illness: A postortem morphometric study. World J Biol Psychiatry (2010) 11(2 Pt 2):282–92. 10.3109/15622970902806124 19360540

[56] O’Donovan SM Sullivan C Koene R Devine E Hasselfeld K Moody CL Cell-subtype-specific changes in adenosine pathways in schizophrenia. Neuropsychopharmacology (2018) 43(8):1667–74. 10.1038/s41386-018-0028-6 PMC6006250 29483661

[57] Konopaske G Dorph-Petersen K Sweet R Pierri J Zhang W Sampson A Effect on chronic antipsychotic exposure on astrocyte and oligodendrocyte numbers in macaque monkeys. Biol Psychiatry (2008) 63:759–65. 10.1016/j.biopsych.2007.08.018 PMC2386415 17945195

[58] Dillon R Hargreaves A Anderson-Schmidt H Castorina M Corvin A Fitzmaurice B Adherence to a Low-Support Cognitive Remediation Training Program for Psychosis. J Nerv Ment Dis (2016) 204(10):741–5. 10.1097/NMD.0000000000000557 27385473

