# Supplementary material for: Free Thyroxine Concentrations Moderate the Response to a Cognitive Remediation Therapy in People With Early Psychosis: A Pilot Randomized Clinical Trial
Source: Front Psychiatry. 2020 Jul 7;11:636. doi: 10.3389/fpsyt.2020.00636 (PMC7358368; doi:10.3389/fpsyt.2020.00636)
Supplement: Supplementary file 1 [file DataSheet_1.pdf]

Supplementary Material.

List S1. CANTAB Cognitive tasks and definition of variables.

1. **Attention Switching Task (AST)**

a. Definition: Attention Switching task has been recently renamed as Multitasking Test (MTT), which is a test of the participant's ability to manage conflicting information provided by the direction of an arrow and its location on the screen and to ignore task-irrelevant information.

b. Administration time: 8 minutes

c. Task format: The test displays an arrow which can appear on either side of the screen (right or left) and can point in either direction (to the right or to the left).
Each trial displays a cue at the top of the screen that indicates to the participant whether they have to select the right or left button according to the "side on which the arrow appeared" or the "direction in which the arrow was pointing".
In some sections of the task this rule is consistent across trials (single task) while in others it may change from trial to trial in a randomized order (multitasking). Using both rules in a flexible manner places a higher demand on cognition than using a single rule.
Some trials display congruent stimuli (e.g. arrow on the right side pointing to the right) whereas other trials display incongruent stimuli, which require a higher cognitive demand (e.g. arrow on the right side of the screen pointing to the left).

d. Outcome measures: Outcome measures for the Multitasking Test include response latencies and error scores that reflect the participant's ability to manage multitasking and the interference of incongruent task-irrelevant information on task performance (i.e. a Stroop-like effect).

e. Creators website: https://www.cambridgecognition.com/cantab/cognitive-tests/executive-function/multitasking-test-mtt

f. CANTAB Variables:

   i. **ASTLCMD**(AST Median-congruent): the median latency of response (from stimulus appearance to button press) on congruent trials.

   ii. **ASTLSWMD** (AST Median-switching):The median latency of response (from stimulus appearance to button press) in assessed block(s) in which the rule is switching.

2. **One Touch Stockings of Cambridge (OTS)**

a. Definition:One Touch Stockings of Cambridge is a test of executive function, based upon the Tower of Hanoi test. It assesses both the spatial planning and the working memory subdomains.

b. Administration time: 10 minutes

c. Task format: The participant is shown two displays containing three colored balls. The displays are presented in such a way that they can be easily perceived as stacks of colored balls held in stockings or socks suspended from a beam. This arrangement makes the 3-D concepts involved apparent to the participant and fits with the verbal instructions. There is a row of numbered

boxes along the bottom of the screen. The test administrator first demonstrates to the participant how to move the balls in the lower display to copy the pattern in the upper display and completes one demonstration problem, where the solution requires one move. The participant must then complete three further problems, one each requiring two moves, three moves and four moves. Next the participant is shown further problems and must work out in their head how many moves the solutions require and then select the appropriate box at the bottom of the screen to indicate their response.

d. Outcome measures: Outcome measures include the number of problems solved on first choice, mean choices to correct, mean latency (speed of response) to first choice and mean latency to correct. Each of these measures may be calculated for all problems, or for problems with a specified number of moves (one-move to five or six moves).
e. Creators website: https://www.cambridgecognition.com/cantab/cognitive-tests/executive-function/one-touch-stockings-of-cambridge-ots
f. CANTAB Variables:
    i. **OTSMDLFC** (OTS Median Latency of First Choice): The median latency, measured from the appearance of the stocking balls until the first box choice was made by the subject. Calculated across all assessed trials where the subject's first response was correct.
    ii. **OTSPSFC** (OTS Problems Solved on First Choice): The total number of assessed trials where the subject chose the correct answer on their first attempt. Calculated across all assessed trials.

3. Paired Associates Learning (PAL):

a. Definition: Paired Associates Learning assesses visual memory and new learning.
b. Administration time: 8 minutes
c. Task format: Boxes are displayed on the screen and are "opened" in a randomized order. One or more of them will contain a pattern. The patterns are then displayed in the middle of the screen, one at a time and the participant must select the box in which the pattern was originally located. If the participant makes an error, the boxes are opened in sequence again to remind the participant of the locations of the patterns. Increased difficulty levels can be used to test high-functioning, healthy individuals.
d. Outcome measures: Outcome measures include the errors made by the participant, the number of trials required to locate the pattern(s) correctly, memory scores and stages completed.
e. Creators website: https://www.cambridgecognition.com/cantab/cognitive-tests/memory/paired-associates-learning-pal
f. CANTAB Variables:

i. **PALFAMS** (PAL First Attempt Memory Score): The number of times a subject chose the correct box on their first attempt when recalling the pattern locations. Calculated across all assessed trials.

ii. **PALTEA** (PAL Total Errors Adjusted): The number of times the subject chose the incorrect box for a stimulus on assessment problems (PALTE), plus an adjustment for the estimated number of errors they would have made on any problems, attempts and recalls they did not reach. This measure allows you to compare performance on errors made across all subjects regardless of those who terminated early versus those completing the final stage of the task.

4. Reaction Time (RTI):

a. Definition:Reaction Time provides assessments of motor and mental response speeds, as well as measures of movement time, reaction time, response accuracy and impulsivity.

b. Administration time: 3 minutes

c. Task format:The participant must select and hold a button at the bottom of the screen. Circles are presented above (one for the simple mode, and five for the five-choice mode.) In each case, a yellow dot will appear in one of the circles, and the participant must react as soon as possible, releasing the button at the bottom of the screen, and selecting the circle in which the dot appeared.

d. Outcome measures:Outcome measures are divided into reaction time and movement time for both the simple and five-choice variants.

e. Creators website: https://www.cambridgecognition.com/cantab/cognitive-tests/attention/reaction-time-rti/

f. CANTAB variables:

i. **RTIFMDMT**(RTI Median Five-Choice Movement Time):The median time taken for a subject to release the response button and select the target stimulus after it flashed yellow on screen.

ii. **RTIFMDRT**(RTI Median Five-Choice Reaction Time): The median duration it took for a subject to release the response button after the presentation of a target stimulus. Calculated across correct, assessed trials in which the stimulus could appear in any one of five locations. Measured in milliseconds.

iii. **RTISMDMT**: RTI Simple Median Movement Time: The median time taken for a subject to release the response button and select the target stimulus after it flashed yellow on screen. Calculated across correct, assessed trials in which the stimulus could appear in one location only. Measured in milliseconds.

iv. **RTISMDRT**: RTI Simple Median Reaction Time: The median duration it took for a subject to release the response button after the presentation of a target stimulus. Calculated across correct, assessed trials in which the stimulus could appear in one location only. Measured in milliseconds.

5. **Rapid Visual Information Processing (RVP)**

a. Definition: Rapid Visual Information Processing is a measure of sustained attention.

b. Administration time: 7 minutes.

c. Task format: A white box is shown in the center of the screen, inside which digits from 2 to 9 appear in a pseudo-random order, at the rate of 100 digits per minute. Participants are requested to detect target sequences of digits (for example, 2-4-6, 3-5-7, 4-6-8). When the participant sees the target sequence they must respond by selecting the button in the center of the screen as quickly as possible. The level of difficulty varies with either one- or three-target sequences that the participant must watch for at the same time.

d. Outcome measures: cover latency (speed of response), probability of false alarms and sensitivity.

e. Creators website: https://www.cambridgecognition.com/cantab/cognitive-tests/attention/rapid-visual-information-processing-rvp

f. Cognitive domains:

   i. **RVPA:**RVP A prime: is the signal detection measure of a subject's sensitivity to the target sequence (string of three numbers), regardless of response tendency (the expected range is 0.00 to 1.00; bad to good). In essence, this metric is a measure of how good the subject is at detecting target sequences.

   ii. **RVPMDL:** RVP Median Response Latency: The median response latency on trials where the subject responded correctly. Calculated across all assessed trials.

   iii. **RVPPFA:** RVP Probability of False Alarm: The number of sequence presentations that were false alarms divided by the number of sequence presentations that were false alarms plus the number of sequence presentations that were correct rejections: (False Alarms/(False Alarms + Correct Rejections)). So the lower the better cognitive performance.

6. **Emotion Recognition Task (ERT)**

a. Definition: The Emotion Recognition Task measures the ability to identify six basic emotions in facial expressions along a continuum of expression magnitude.

b. Administration time: 6-10 minutes.

c. Task format: Computer-morphed images derived from the facial features of real individuals, each showing a specific emotion, are displayed on the screen, one at a time. Each face is displayed for 200ms and then immediately covered up to prevent residual processing of the image. The participant must select which emotion the face displayed from 6 options (sadness, happiness, fear, anger, disgust or surprise).

d. Outcome measures: The outcome measures for ERT cover percentages and numbers correct or incorrect and overall response latencies, which can be looked at either across individual emotions or across all emotions at once.

e. Creators website:https://www.cambridgecognition.com/cantab/cognitive-tests/emotion-and-social/emotion-recognition-task-ert

f. Cognitive domains:

   i. **ERTOMDRT** (ERT Overall Median Reaction Time): The overall median latency for a subject to select an emotion word after being presented with a stimulus. Calculated across all assessed trials.

ii. **ERTTH** (ERT Total Hits): The total number of correct responses (emotion selection) the subject made across all assessed trials.

7. Spatial Working Memory (SWM)

a. Definition:Spatial Working Memory requires retention and manipulation of visuospatial information. This self-ordered test has notable executive function demands and provides a measure of strategy as well as working memory errors.
b. Administration time: 4 minutes.
c. Task format: The test begins with a number of coloured squares (boxes) shown on the screen. The aim of this test is that by selecting the boxes and using a process of elimination, the participant should find one yellow ‘token’ in each of a number of boxes and use them to fill up an empty column on the right-hand side of the screen. Depending on the difficulty level used for this test, the number of boxes can be gradually increased until a maximum of 12 boxes are shown for the participants to search. The colour and position of the boxes used are changed from trial to trial to discourage the use of stereotyped search strategies.
d. Outcome measures: include errors (selecting boxes that have already been found to be empty and revisiting boxes which have already been found to contain a token) and strategy.
e. Creators website: https://www.cambridgecognition.com/cantab/cognitive-tests/executive-function/spatial-executive-function-swm
f. Cognitive domains:
   i. **SWMBE** (SWM Between Errors): The number of times the subject incorrectly revisits a box in which a token has previously been found. Calculated across all assessed four, six and eight token trials.
   ii. **SWMBE4** (SWM Between errors 4 boxes): The number of times a subject revisits a box in which a token has previously been found. Calculated across all trials with 4 tokens only.
   iii. **SWMBE6** (SWM Between errors 6 boxes: The number of times the subject revisits a box in which a token has previously been found. Calculated across all trials with 6 tokens only.
   iv. **SWMBE8** (SWM Between errors 8 boxes: The number of times the subject revisits a box in which a token has previously been found. Calculated across all trials with 8 tokens only.
   v. **SWMS** (SWM Strategy (6-8 boxes)): The number of times a subject begins a new search pattern from the same box they started with previously. If they always begin a search from the same starting point we infer that the subject is employing a planned strategy for finding the tokens. Therefore, a low score indicates high strategy use (1 = they always begin the search from the same box), a high score indicates that they are beginning their searches from many different boxes. Calculated across assessed trials with 6 tokens or more.

8. Verbal Recognition Memory (VRM)

a. Definition: Verbal Recognition Memory assesses verbal memory and new learning. It measures the ability to encode and subsequently retrieve verbal information, with recall tapping into fronto-temporal networks and recognition assessing hippocampal areas.
b. Administration time: 10 minutes.

c. Task format: The participant is shown a sequence of words on screen one by one. The participant is then tasked with recalling the words, whilst a rater marks which ones they remembered. In the next phase, the participant is presented with two words, one from the original list and one distractor and is asked to choose which one they have seen before, in a 2-force choice paradigm. The latter recognition phase is then repeated after a delay.
d. Outcome measures: Outcome measures include the number of distinct words for the free-recall phase and number of correct and incorrect responses for the immediate and delayed recognition parts of the test.
e. Creators website: https://www.cambridgecognition.com/cantab/cognitive-tests/memory/verbal-recognition-memory-vrm
f. Cognitive domains:
    a. **VRMDRTC** (VRM Delayed Recognition: Total Correct): The total number of target words that the subject correctly recognizes in the delayed recognition phase, plus the total number of distractor words that the subject correctly rejects.
    b. **VRMFRDS** (VRMDRCRS + VRMDRCRD: VRM Free Recall Distinct Stimuli: The total number of distinct words that are correctly recalled from the presentation phase by the subject during the immediate free recall stage.
    c. **VRMIRTC** (VRM Immediate Recognition: Total Correct): The total number of target words that the subject correctly recognises, plus the total number of distractor words that the subject correctly rejects. (VRMIRCRS + VRMIRCRD).

Figure S1. Flow chart of participants of the study.

Abbreviations: CRT= Cognitive rehabilitation therapy; TAU= Treatment as usual.

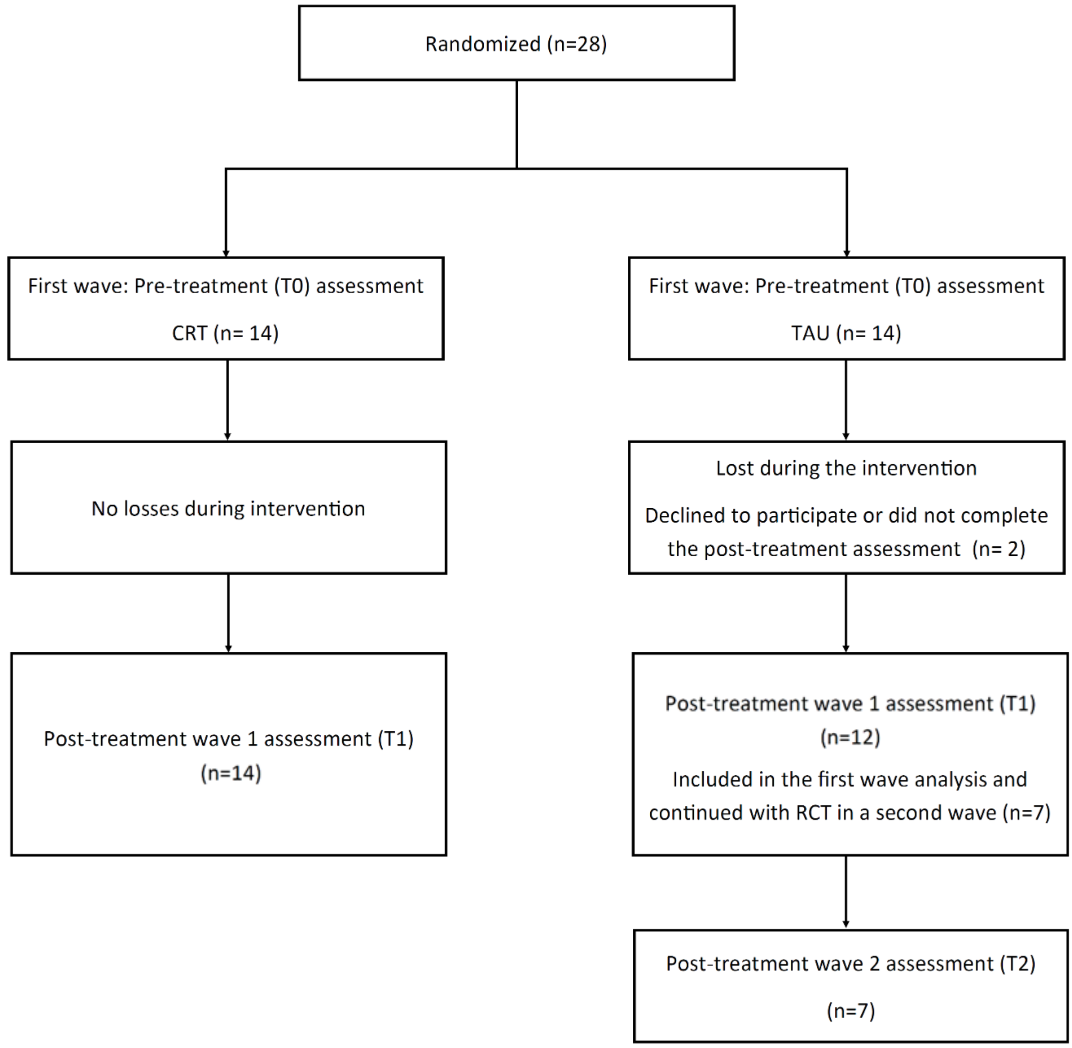

Figure S2. Longitudinal changes in spatial working memory errors (6 tokens) after the cognitive remediation therapy by free thyroxine concentrations at the baseline visit.

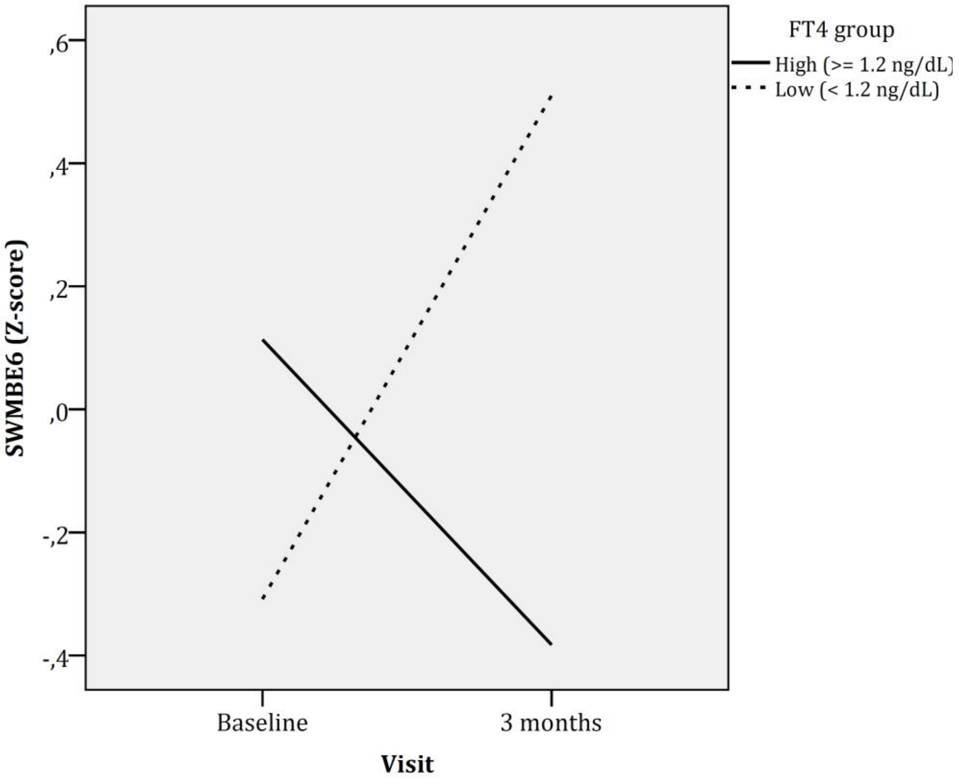

Figure S3. Longitudinal changes in verbal memory (delayed recall) after cognitive remediation therapy by free thyroxine concentrations at the baseline visit.

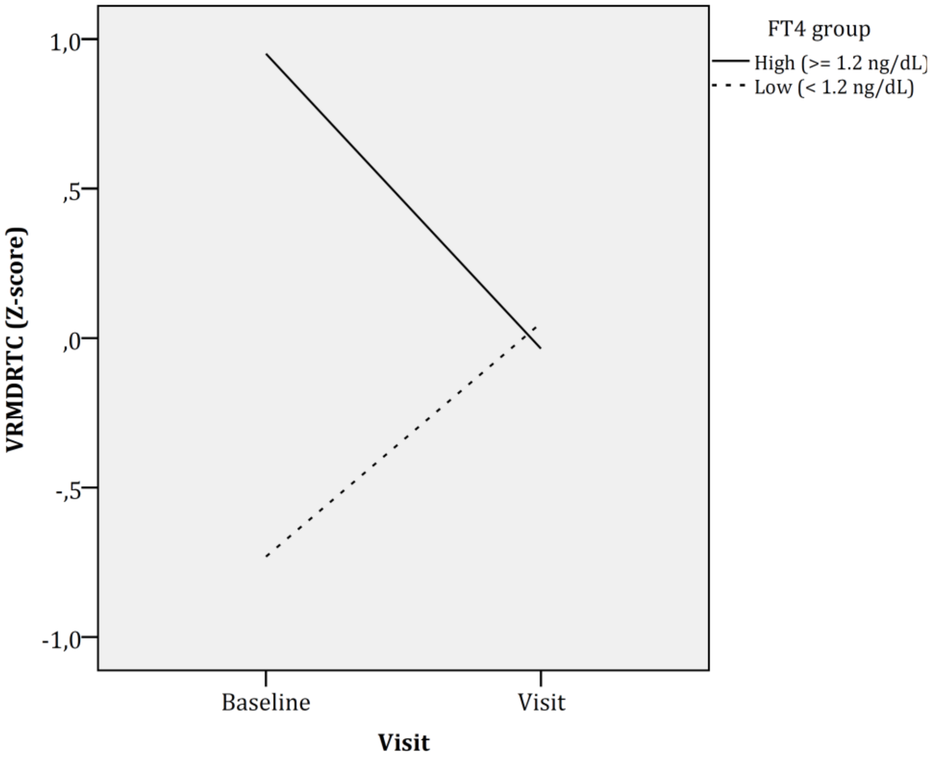

Table S1. Results of the linear mixed models exploring longitudinal changes in cognition in the first wave (CRT vs TAU). Estimated fixed effects (calculated with z-scores) for each variable and the tested interactions between variables are shown.

**CANTAB variable[1]**	**Time**			**CRT**			**FT4**			**TSH**			**Time x CRT**			**Time x FT4**			**CRT x FT4**			**Time x CRT x FT4**		
	Effect	SE	p	Effect	SE	p	Effect	SE	p	Effect	SE	p	Effect	SE	p	Effect	SE	p	Effect	SE	p	Effect	SE	p
**ASTLCMD***	-0.41	0.28	0.158	0.10	0.34	0.775	0.01	0.15	0.964	0.03	0.10	0.730	**0.96**	**0.41**	**0.033**	-0.08	0.37	0.835	-0.05	0.20	0.812	0.50	0.48	0.319
**ASTLSWMD***	**-0.66**	**0.27**	**0.032**	-0.33	0.40	0.419	-0.15	0.20	0.458	0.01	0.13	0.929	**1.04**	**0.40**	**0.023**	0.03	0.39	0.932	0.32	0.27	0.257	0.08	0.54	0.878
**OSTMDLFC***	-0.59	0.30	0.072	0.12	0.39	0.751	0.01	0.20	0.951	0.02	0.14	0.883	0.61	0.44	0.190	0.14	0.43	0.754	0.22	0.27	0.432	0.10	0.58	0.863
**OTSPSFC**	0.19	0.41	0.654	0.67	0.29	0.031	**0.61**	**0.16**	**0.001**	0.26	0.13	0.054	-0.66	0.60	0.288	0.11	0.59	0.851	**-0.57**	**0.23**	**0.021**	-0.27	0.78	0.735
**PALFAMS**	0.55	0.28	0.062	-0.23	0.37	0.537	**0.50**	**0.21**	**0.026**	**0.38**	**0.12**	**0.004**	**-1.16**	**0.40**	**0.010**	0.03	0.34	0.922	-0.55	0.31	0.084	-0.43	0.47	0.377
**PALTEA***	-0.65	0.32	0.062	-0.05	0.39	0.907	-0.45	0.23	0.055	**-0.40**	**0.12**	**0.004**	0.92	0.47	0.065	-0.10	0.37	0.787	0.25	0.34	0.459	0.64	0.51	0.220
**RTIFMDMT***	-0.24	0.34	0.496	0.26	0.35	0.459	0.16	0.18	0.399	0.04	0.14	0.786	**1.20**	**0.49**	**0.031**	-0.13	0.48	0.788	-0.27	0.26	0.312	1.12	0.65	0.107
**RTIFMDRT***	-0.27	0.40	0.503	0.43	0.45	0.349	-0.26	0.25	0.300	-0.05	0.18	0.775	0.53	0.57	0.373	0.18	0.54	0.745	0.22	0.36	0.545	0.21	0.75	0.785
**RTISMDMT***	-0.04	0.37	0.924	0.16	0.40	0.698	-0.14	0.21	0.515	0.07	0.16	0.666	0.28	0.56	0.629	0.61	0.52	0.261	0.08	0.31	0.786	-0.38	0.71	0.602
**RTISMDRT***	0.04	0.42	0.934	0.54	0.47	0.257	-0.14	0.26	0.596	0.00	0.18	0.995	-0.08	0.63	0.906	0.17	0.57	0.775	-0.29	0.37	0.450	0.29	0.79	0.724
**RVPA**	0.03	0.34	0.928	-0.10	0.27	0.706	0.18	0.14	0.212	0.15	0.11	0.197	-0.12	0.50	0.815	-0.20	0.48	0.684	-0.11	0.20	0.603	-0.25	0.64	0.703
**RVPMDL***	-0.11	0.38	0.788	-0.05	0.41	0.906	-0.19	0.22	0.397	-0.20	0.16	0.220	0.09	0.56	0.871	0.40	0.53	0.461	0.42	0.33	0.212	-0.47	0.72	0.522
**RVPPFA***	-0.32	0.62	0.617	-0.14	0.30	0.635	-0.28	0.17	0.101	-0.24	0.14	0.093	1.19	0.89	0.201	0.08	0.86	0.928	0.16	0.25	0.521	0.75	1.13	0.521
**ERTOMDRT***	0.08	0.51	0.874	0.49	0.26	0.075	0.03	0.14	0.855	-0.08	0.12	0.523	-0.25	0.74	0.737	0.14	0.72	0.853	0.11	0.21	0.615	-0.73	0.96	0.457
**ERTTH**	-0.40	0.32	0.244	-0.53	0.34	0.133	0.04	0.19	0.847	0.19	0.14	0.176	0.77	0.47	0.125	-0.21	0.44	0.637	-0.12	0.28	0.668	-0.02	0.61	0.971
**SWMBE***	-0.46	0.32	0.165	-0.20	0.46	0.661	-0.17	0.27	0.543	-0.08	0.15	0.604	0.45	0.47	0.351	0.11	0.44	0.810	0.30	0.38	0.439	0.14	0.62	0.819
**SWMBE4***	-0.44	0.28	0.139	0.27	0.45	0.557	-0.27	0.24	0.262	0.12	0.15	0.422	-0.16	0.41	0.699	-0.10	0.39	0.803	-0.04	0.33	0.895	0.45	0.55	0.427
**SWMBE6***	**-0.97**	**0.36**	**0.018**	-0.63	0.47	0.186	0.09	0.26	0.731	-0.10	0.17	0.559	**1.77**	**0.52**	**0.004**	-0.48	0.46	0.322	0.11	0.38	0.766	1.05	0.65	0.124
**SWMBE8***	-0.04	0.38	0.926	0.02	0.47	0.960	-0.28	0.28	0.338	-0.11	0.17	0.540	-0.37	0.57	0.524	0.19	0.51	0.715	0.39	0.42	0.361	-0.28	0.72	0.700
**SWMS***	-0.04	0.52	0.936	-0.25	0.49	0.618	0.00	0.29	0.989	-0.07	0.19	0.727	0.39	0.77	0.620	0.01	0.65	0.990	0.05	0.45	0.911	0.35	0.90	0.700
**VRMDRTC**	-0.93	0.46	0.059	-0.74	0.38	0.064	0.20	0.21	0.349	-0.01	0.16	0.949	1.08	0.66	0.119	-0.66	0.61	0.292	0.21	0.32	0.519	0.57	0.81	0.495
**VRMFRDS**	-0.13	0.36	0.732	-0.69	0.44	0.133	0.21	0.26	0.409	-0.06	0.14	0.676	0.17	0.52	0.751	0.55	0.41	0.195	-0.20	0.38	0.597	-0.11	0.57	0.852
**VRMIRTC**	-0.36	0.42	0.405	**-1.28**	**0.34**	**0.001**	0.16	0.19	0.429	-0.15	0.13	0.286	**1.27**	**0.60**	**0.048**	-0.43	0.52	0.419	0.32	0.29	0.274	0.73	0.67	0.293

Abbreviations: CRT, Cognitive remediation therapy; TAU, Treatment as usual; SE, Standard error; FT4, Free thyroxin; TSH, Thyroid-stimulating hormone.

[1]For all cognitive tasks, lower scores reflect better cognitive performance unlike OTSPSFC, PALFAMS, RVPA, ERTTH, and all VRM tasks (which are the opposite; higher scores reflect better cognitive performance). Reverse scores have been marked with an asterisk (*), indicating that higher scores reflect worse cognitive performance.
